# Correction Notice: Dual-sided Voltage-sensitive Dye Imaging of Leech Ganglia

**DOI:** 10.21769/BioProtoc.2985

**Published:** 2018-08-05

**Authors:** Yusuke Tomina, Daniel A. Wagenaar

**Affiliations:** Division of Biology and Biological Engineering, California Institute of Technology, Pasadena, United States

Thank you very much for dealing with our protocol issued on March 5, 2018 (https://bio-protocol.org/e2751).

We have noticed a simple, but a very problematic typo in a recipe of physiological saline in the protocol. In the Recipe section of the protocol, the concentration of CaCl_2_ is "8 mM" as below, but this is completely wrong. Correct concentration of CaCl_2_ is "1.8 mM". We are sorry that we had not noticed this typo when we submitted a manuscript. We would appreciate it if you could make a correction only to this part ("8" → "1.8").

## 
References



Tomina, Y. and Wagenaar, D. A. (2018).  Dual-sided Voltage-sensitive Dye Imaging of Leech Ganglia. 
*Bio-protocol* 8(5): e2751.


